# The PTS^Ntr^-KdpDE-KdpFABC Pathway Contributes to Low Potassium Stress Adaptation and Competitive Nodulation of Sinorhizobium fredii

**DOI:** 10.1128/mbio.03721-21

**Published:** 2022-05-02

**Authors:** Xue-Ying Feng, Yu Tian, Wen-Jing Cui, Yue-Zhen Li, Dan Wang, Yanbo Liu, Jian Jiao, Wen-Xin Chen, Chang-Fu Tian

**Affiliations:** a State Key Laboratory of Agrobiotechnology, and College of Biological Sciences, China Agricultural University, Beijing, China; b MOA Key Laboratory of Soil Microbiology, and Rhizobium Research Center, China Agricultural University, Beijing, China; c High School Affiliated to Renmin University, Beijing, China; Philipps University Marburg; University of Würzburg

**Keywords:** legume, potassium, soybean, symbiosis

## Abstract

The rhizobium-legume symbiosis is essential for sustainable agriculture by reducing nitrogen fertilizer input, but its efficiency varies under fluctuating soil conditions and resources. The nitrogen-related phosphotransferase system (PTS^Ntr^) consisting of PtsP, PtsO, and PtsN is required for optimal nodulation and nitrogen fixation efficiency of the broad-host-range Sinorhizobium fredii CCBAU45436 associated with diverse legumes, though the underlying mechanisms remain elusive. This work characterizes the PtsN-KdpDE-KdpFABC pathway that contributes to low potassium adaptation and competitive nodulation of CCBAU45436. Among three PtsN, PtsN_1_ is the major functional homolog. The unphosphorylated PtsN_1_ binds the sensory kinase KdpD through a non-canonical interaction with the GAF domain of KdpD, while the region covering HisKA-HATPase domains mediates the interaction of KdpD with the response regulator KdpE. KdpE directly activates the *kdpFABC* operon encoding the conserved high-affinity potassium uptake system. Disruption of this signaling pathway leads to reduced nodule number, nodule occupancy, and low potassium adaptation ability, but without notable effects on rhizoplane colonization. The induction of key nodulation genes *NIN* and *ENOD40* in host roots during early symbiotic interactions is impaired when inoculating the *kdpBC* mutant that shows delayed nodulation. The nodulation defect of the *kdpBC* mutant can be rescued by supplying replete potassium. Potassium is actively consumed by both prokaryotes and eukaryotes, and components of the PTS^Ntr^-KdpDE-KdpFABC pathway are widely conserved in bacteria, highlighting the global importance of this pathway in bacteria-host interactions.

## INTRODUCTION

Protein phosphorylation is one of the major mechanisms underlying organisms’ adaptation to fluctuating conditions and resources in various ecological niches. Bacterial kinases can be classified into four major families (1). The eukaryote-like protein kinases (also referred to Hanks-type kinases) phosphorylate a large spectrum of substrates at their serine and threonine residues (2). The BY kinases catalyze phosphorylation of targets at tyrosine residues (3). The two-component systems include a sensory histidine kinase that autophosphorylates at a conserved histidine by using the γ-phosphoryl group of ATP, and a response regulator that receives the phosphoryl from the histidine-phosphorylated kinase at a conserved aspartate residue (4). The fourth family is the phosphotransferase system harboring a group of enzymes that sequentially transfer the phosphoryl group derived from phosphoenolpyruvate to a histidine residue of downstream members of the system (5). The canonical phosphotransferase system (PTS) directly involved in carbohydrate uptake (6, 7) and the nitrogen-related phosphotransferase system (PTS^Ntr^) have been found in various bacteria. Both canonical PTS and PTS^Ntr^ have enzyme I (EI or EI^Ntr^), histidine protein (HPr or NPr), and enzyme II (EIIA or EIIA^Ntr^), while the PTS^Ntr^ lacks substrate specific EIIB and EIIC required for carbohydrate uptake (5). The PTS^Ntr^ is characterized by its regulatory roles in diverse processes such as the metabolism of nitrogen and carbon, phosphate starvation, and K^+^ homeostasis (5, 8, 9).

K^+^ selective cation channels are essential for both prokaryotes and eukaryotes to maintain the asymmetric K^+^/Na^+^ distribution, with K^+^ as the major cation in the cytoplasm while Na^+^ being dominant in the media (10). Bacteria usually harbor a variable number of K^+^ uptake systems including Trk, Ktr, Kup, and Kdp reflecting adaptations to different niches (11, 12). The H^+^-dependent Trk and Na^+^-dependent Ktr show low cation selectivity with moderate binding affinity, while the K^+^ uptake permease Kup and the P-type ATPase mediating system Kdp are considered specific K^+^ transporters with Kdp being the high-affinity K^+^ transporter (11, 12). Moreover, *kdp* genes are inducible under low K^+^ conditions where the sensor kinase KdpD phosphorylates the response regulator KdpE that promotes transcription of the *kdpFABC* operon (13, 14). Although the precise signal recognized by the membrane-bound KdpD is still under discussion (14), it has been demonstrated that unphosphorylated EIIA^Ntr^ can interact with KdpD in Escherichia coli, Rhizobium leguminosarum, and Pseudomonas putida and activates the transcription of *kdpFABC* genes (15–17). With evidences from mutants of K^+^ uptake systems of Salmonella (18), Staphylococcus aureus (19), Helicobacter pylori (20), Mycobacterium tuberculosis (21), Pectobacterium wasabiae (22), Streptococcus mutans (23), and Sinorhizobium meliloti (24), it is only just emerging that K^+^ is an environmental cue and a key player in host-bacteria interactions (25, 26).

These studies imply that a PTS^Ntr^-KdpDE-KdpFABC pathway might be involved in host-bacteria interactions, though not fully established in any individual system yet. To test this hypothesis, we focused on the mutualistic interactions between rhizobia and legumes which innovate root nodules where rhizobia reduce atmospheric nitrogen into ammonia to support plant growth (27, 28). Our previous work reveals that PTS^Ntr^ is essential for effective symbiosis of Sinorhizobium fredii CCBAU45436 with soybean and pigeon pea plants (29). The symbiotic defects of mutants lacking EI^Ntr^ (*ptsP*) or Npr (*ptsO*) can be partially rescued by further deletion of an EIIA^Ntr^ (*ptsN_1_*) while the single *ptsN_1_* mutant is indistinguishable from the wild-type strain except impaired nodulation and nodule occupancy abilities (29). In this work, we aimed to characterize the EIIA^Ntr^-KdpDE-KdpFABC pathway in CCBAU45436, and investigate the potential role of Kdp in symbiotic interactions. Three PtsN homologs were characterized for their effect on symbiosis and low potassium adaptation, and ability to interact with KdpD. Distinct domains of KdpD involved in interactions with the major EIIA^Ntr^ (PtsN_1_) and KdpE were identified, and direct activation of the *kdpFABC* operon by KdpE was demonstrated. The effects of phosphorylated or unphosphorylated PtsN_1_ and the downstream KdpDE-KdpFABC pathway in low potassium adaptation and symbiotic interactions were further characterized. Together with the transcriptional analysis of key nodulation genes of soybean plants during early interaction stages and nodulation kinetics assay, the important role of K^+^ uptake in optimal nodulation mediated by the PTS^Ntr^-KdpDE-KdpFABC pathway is proposed and discussed.

## RESULTS AND DISCUSSION

### Expansion of PtsN homologs in *Rhizobiaceae*.

**FIG 1:**
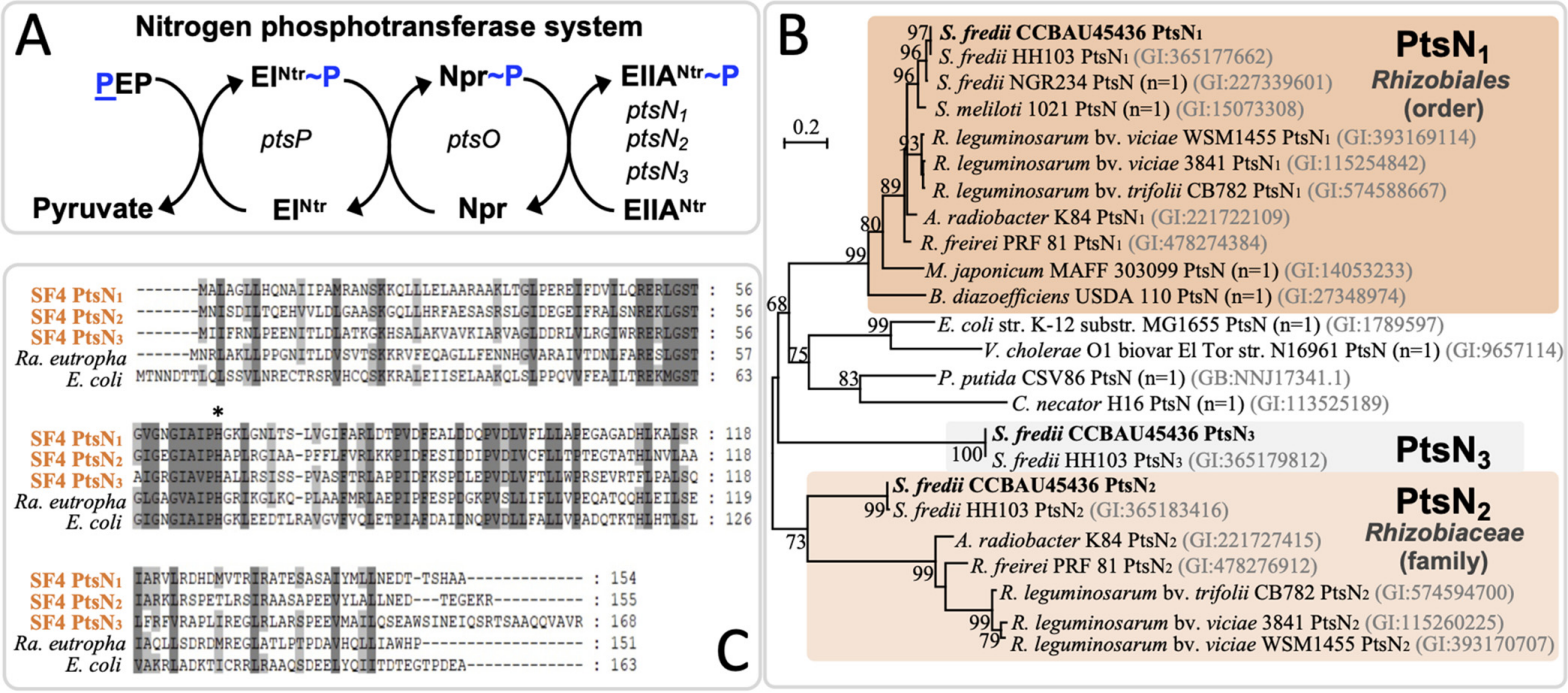
PtsN homologs in Sinorhizobium fredii CCBAU45436. (A) Components of PTS^Ntr^ in *S. fredii* CCBAU45436 (SF4) including *ptsP* encoding EI^Ntr^, *ptsO* encoding Npr, and three copies of *ptsN* encoding EIIA^Ntr^. (B) The unrooted maximum likelihood phylogenetic tree of EIIA^Ntr^ homologs from representative bacterial species. Bootstrap values above 60 are shown. *n* = 1 indicates that only one EIIA^Ntr^ can be identified in the corresponding strain. *R*, *Rhizobium*; *S*, *Sinorhizobium*; *A*, *Agrobacterium*; *M*, *Mesorhizobium*; *B*, *Bradyrhizobium*; *E*, Escherichia; *V*, *Vibrio*; *P*, Pseudomonas; *C*, *Cupriavidus*. (C) Alignment of PtsN homologs showing the conserved histidine (*, H66 in SF4 EIIA^Ntr^) involved in phosphorylation (~P). PtsN homologs of Ralstonia eutropha H16 and E. coli MG1655 are included for comparison.

The regulatory roles of PTS^Ntr^ have been intensively studied in various pathogens, with PtsN being the major output regulator (5). The broad-host-range rhizobium *S. fredii* CCBAU45436 (SF4 hereafter) (30–32) has three PtsN homologs (Fig. 1A). Phylogenetic analysis indicated their distinct phyletic distribution (Fig. 1B), with PtsN_1_ conserved in the *Rhizobiales* order (including genera *Rhizobium*, *Sinorhizobium*, *Agrobacterium*, *Mesorhizobium*, and *Bradyrhizobium*) and clustered with PtsN from other bacteria in a highly supported orthologous group, with PtsN_2_ present in some species of the *Rhizobiaceae* family, and with PtsN_3_ identified in *S. fredii* strains SF4 and HH103. Although PtsN_1_, PtsN_2_, and PtsN_3_ of SF4 belong to three separate clusters (Fig. 1B), sequence alignment analysis (Fig. 1C) showed that they have the conserved histidine residue (H66) which is the only phosphorylated site of EIIA^Ntr^ homologs as demonstrated previously (33). To investigate the potential role of the three *ptsN* homologs of SF4 in symbiosis, all single, double and triple in-frame deletion mutants were constructed and tested for their symbiotic performance on soybean plants (Table S1). All test mutants were able to form functional nodules which supported the growth of soybean plants at a similar level as the wild-type SF4 regarding shoot dry weight and leaf chlorophyll content (Table S1; ANOVA followed by Duncan's test, alpha = 0.05). The *ptsN_1_*, *ptsN_12_*, *ptsN_13_*, *ptsN_123_*, and *ptsN_2_* mutants formed similar numbers of nodules which were significantly less than those induced by SF4, *ptsN_3_*, and *ptsN_23_* mutants (ANOVA followed by Duncan's test, alpha = 0.05). This suggests that PtsN homologs are important for optimal nodulation of *S. fredii* on soybean plants, with the more conserved PtsN_1_ being the major functional homolog and the positive effect of PtsN_2_ depending on the presence of PtsN_3_. This is in line with the finding in R. leguminosarum which harbors two PtsN homologs with PtsN_1_ as the major EIIA^Ntr^ (9).

10.1128/mbio.03721-21.1 TABLE S1 Symbiotic performance of *ptsN* mutants on soybean plants. Download Table S1, PDF file, 0.1 MB. Copyright © 2022 Feng et al. 2022 Feng et al. https://creativecommons.org/licenses/by/4.0/ This content is distributed under the terms of the Creative Commons Attribution 4.0 International license.

Notably, a R. leguminosarum mutant lacking functional EIIA^Ntr^ formed a similar number of nodules as the wild-type strain but fixed less nitrogen on pea plants while *S. fredii* lacking EIIA^Ntr^ formed fewer effective nodules on soybean plants (Table S1) (9, 29). This contrasting phenotype may be at least partially due to different stimuli encountered by rhizobia during the establishment and maintenance of the determinate (with transient meristems, such as soybean and cowpea) and indeterminate nodules (with persistent meristems, such as pea and alfalfa) (34, 35). Rhizobia terminally differentiate (enlarged cell size and reduced reproductive ability) in pea and alfalfa nodules but not in soybean and cowpea nodule cells (27, 36–38). Nitrogen-fixing rhizobial cells accumulate more carbon storage polymer poly-β-hydroxybutyrate (PHB) in soybean and cowpea nodules than in pea and alfalfa nodules (27, 36–38). We have revealed that PHB biosynthesis and nitrogen fixation is blocked in the *ptsP* and *ptsO* mutants but restored in the *ptsPN_1_* and *ptsON_1_* double mutants of *S. fredii* in soybean nodules (29). Although the regulation of nitrogen and carbon metabolism by PTS^Ntr^ is supported by evidences from both *S. fredii-*soybean and R. leguminosarum*-*pea symbioses (9, 29), the underlying signaling pathway mediated by PTS^Ntr^ in PHB biosynthesis and other adaptive processes in these contrasting rhizobium-legume pairs remains elusive.

### PtsN_1_ and PtsN_2_ interact with KdpD and contribute to low potassium adaptation and optimal nodulation.

**FIG 2:**
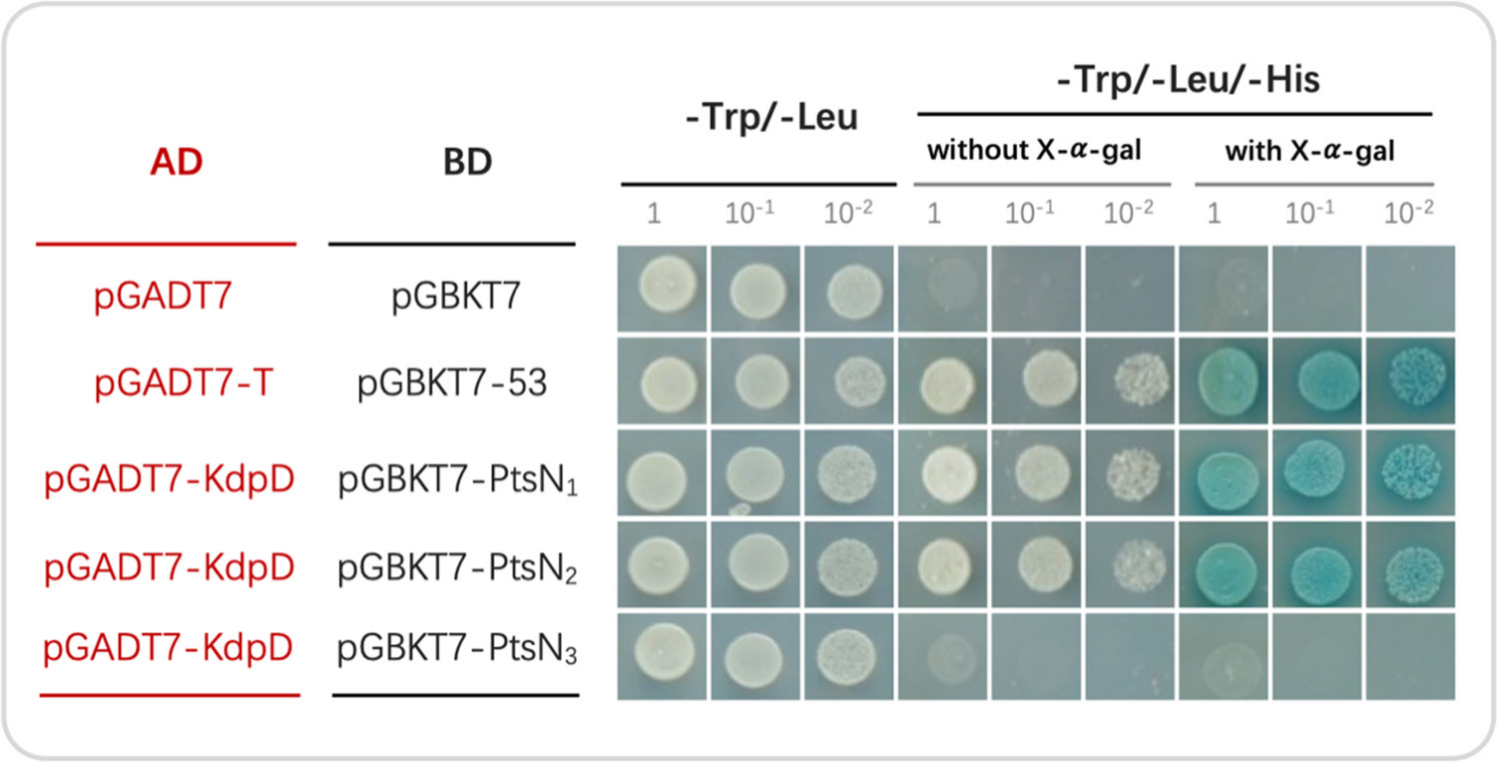
PtsN_1_ and PtsN_2_ directly interact with KdpD. Three dilutions are shown from the yeast two-hybrid experiment with pGADT7/pGBKT7 and pGADT7-T/pGBKT7-53 as negative and positive controls, respectively. Yeast cells were co-transformed with AD and BD vectors. The growth on the medium lacking Trp/Leu/His, and blue color indicate protein interaction.

**FIG 3:**
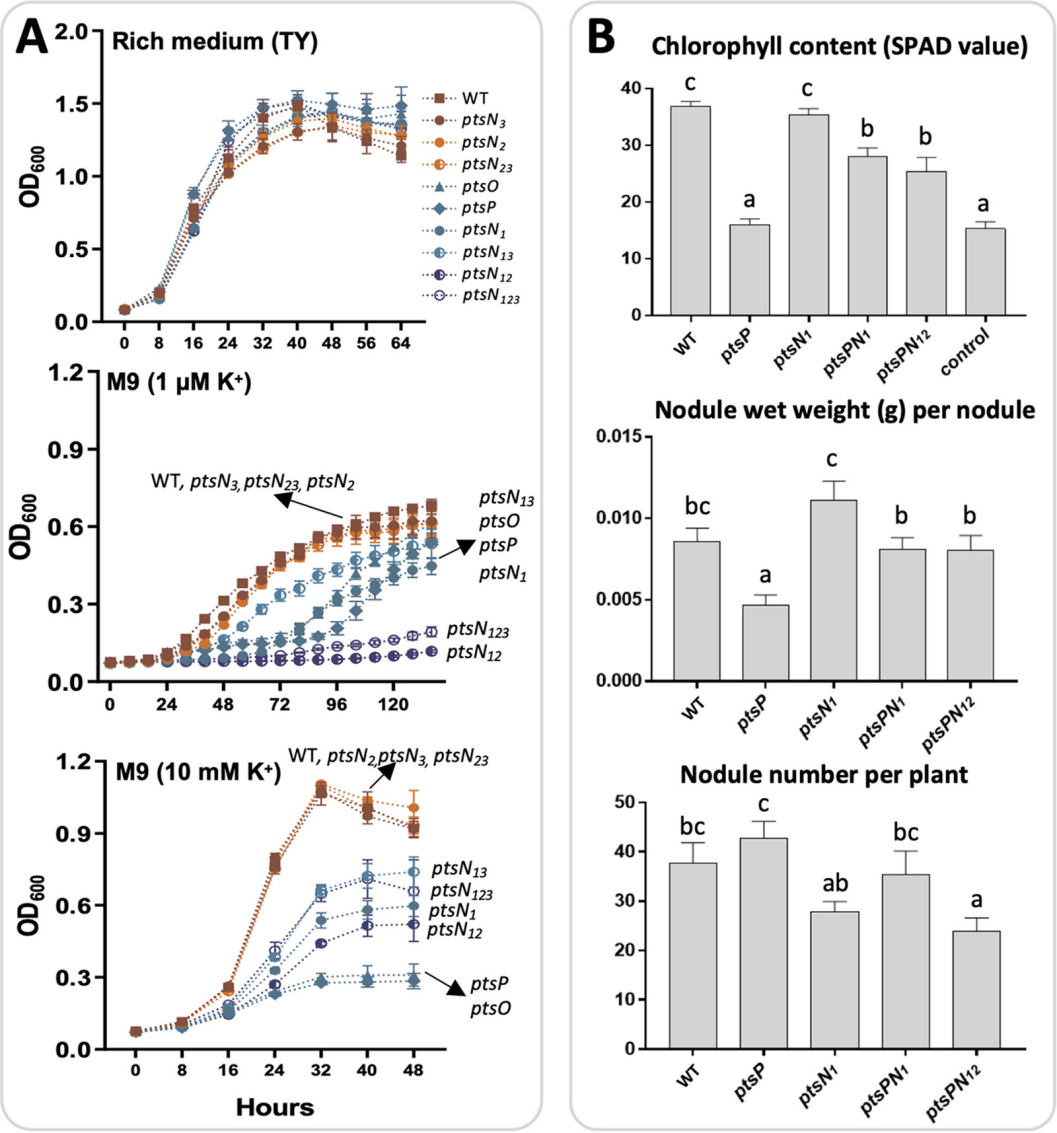
Cumulative role of *ptsN_1_* and *ptsN_2_* in low K^+^ adaptation and nodulation. (A) Growth curves in rich medium and minimum medium supplied with 1 μM and 10 mM K^+^. Results are based on average ± SEM of three biological replicates. (B) Symbiotic performance on soybean plants. Different letters indicate significant difference between treatments (Average ± SEM; ANOVA followed by Duncan’s test, alpha = 0.05). More than eight plants were scored.

As the interaction between EIIA^Ntr^ and KdpD has been recurrently found in E. coli, R. leguminosarum, and Pseudomonas putida (15–17), the yeast two-hybrid experiment was used herein to identify which PtsN homolog(s) may keep this conserved function. It turned out that PtsN_1_ and PtsN_2_ rather than PtsN_3_ interact with KdpD under test conditions (Fig. 2). After an exploring test of different levels of K^+^ (Fig. S1), 1 μM and 10 mM was considered as low and replete K^+^ conditions, respectively. Under the low K^+^ condition (1 μM K^+^), the *ptsN_12_* mutant exhibited a more severe growth defect than the *ptsN_1_* or *ptsN_2_* mutants (Fig. 3A), implying cumulative effects associated with PtsN_1_ and PtsN_2_. Similarly, the *ptsN_123_* mutant grew worse than the *ptsN_13_* mutant that in turn grew worse than the *ptsN_3_* mutant. Noteworthy, growth delay was observed for the *ptsN_1_* mutant but not for the *ptsN_2_*, *ptsN_3_*, and *ptsN_23_* mutants, suggesting PtsN_1_ as the major EIIA^Ntr^. Although the *ptsN_3_* mutant was indistinguishable from SF4, the *ptsN_123_* and *ptsN_13_* mutants grew slightly better than the *ptsN_12_* and *ptsN_1_* mutants, respectively, indicating a potential negative regulatory role of PtsN_3_ in low K^+^ adaptation. On the other hand, the growth rate of all test strains was higher under K^+^ replete condition (10 mM K^+^) than low K^+^ condition (Fig. 3A). PtsN_1_ was required for the maximum growth rate of SF4 under this K^+^ replete condition likely due to its regulatory roles in carbon metabolism (16, 29), with cumulative contribution by PtsN_2_ and antagonistic effect from PtsN_3_. The strong synthetic negative phenotype of the *ptsN_12_* mutant under low K^+^ condition was however not observed under this K^+^ replete condition. Moreover, the *ptsP* and *ptsO* mutants grew faster than the *ptsN_12_* and *ptsN_123_* mutants under low K^+^ condition while the reverse was observed under K^+^ replete condition (Fig. 3A), suggesting a regulatory duality mediated by switching EIIA^Ntr^ phosphorylation status (Fig. 1A) (5). These results, particularly the contrasting growth phenotypes of the *ptsN_12_* mutant under K^+^ replete and deplete conditions, implied that EIIA^Ntr^ is critical for *S. fredii* adaptation to fluctuating levels of K^+^ that is also consumed by other organisms in the same habitat including the interacting eukaryote hosts (25, 26, 39).

10.1128/mbio.03721-21.7 FIG S1 Influence of K^+^ levels on the growth of the *kdpBC*, *kdpDE* and *ptsN_123_* mutants in the minimum medium. Download FIG S1, PDF file, 0.2 MB. Copyright © 2022 Feng et al. 2022 Feng et al. https://creativecommons.org/licenses/by/4.0/ This content is distributed under the terms of the Creative Commons Attribution 4.0 International license.

The *ptsP* mutant has pleiotropic defects including symbiotic inefficiency which can be partially rescued by further deletion of *ptsN_1_* (29). To investigate whether the second KdpD-interacting EIIA^Ntr^ PtsN_2_ has cumulative contribution to symbiotic efficiency, the triple mutant *ptsPN_12_* was constructed. This mutant was as efficient as the *ptsPN_1_* mutant and performed better than the *ptsP* mutant regarding chlorophyll content of inoculated soybean plants (ANOVA followed by Duncan's test, alpha = 0.05), though shoot dry weight was partially recovered in the *ptsPN_1_* and *ptsPN_12_* treatments at an insignificant level under test conditions (Fig. 3B; Table S2). Inefficient nodules induced by the *ptsP* mutant were significantly smaller than those efficient nodules formed by SF4, *ptsN_1_*, *ptsPN_1_* and *ptsPN_12_* mutants (Fig. 3B; Table S2). The *ptsN_1_* and *ptsPN_12_* mutants formed significantly less nodules than the *ptsP* and *ptsPN_1_* mutants, respectively. It seems that the contrasting number and weight of nodules between the *ptsP* and *ptsN_1_* treatments are in line with the canonical model of autoregulation of nodulation (28). However, nodule weight was similar between the *ptsPN_1_* and *ptsPN_12_* treatments (Fig. 3B; Table S2), indicating that the reduced nodule number in the *ptsPN_12_* treatment compared with the *ptsPN_1_* treatment may also be regulated by processes other than autoregulation of nodulation. Therefore, a cumulative role of PtsN_2_ in optimal nodulation was revealed by comparing the *ptsP*, *ptsPN_1_*, and *ptsPN_12_* mutants. Taken together with the nodulation phenotypes and growth curves of various *ptsN* mutants (Table S1; Fig. 3), despite an expansion of PtsN copies in the *Rhizobiaceae* family (Fig. 1B), these findings suggest PtsN_1_ as the major EIIA^Ntr^ in symbiotic interaction and low K^+^ adaptation with a cumulative contribution by PtsN_2_.

10.1128/mbio.03721-21.2 TABLE S2 Symbiotic performance of *ptsPN_1_* and *ptsPN_12_* mutants on soybean plants. Download Table S2, PDF file, 0.1 MB. Copyright © 2022 Feng et al. 2022 Feng et al. https://creativecommons.org/licenses/by/4.0/ This content is distributed under the terms of the Creative Commons Attribution 4.0 International license.

### Optimal nodulation and low potassium adaptation mediated by the EIIA^Ntr^-Kdp pathway.

**FIG 4:**
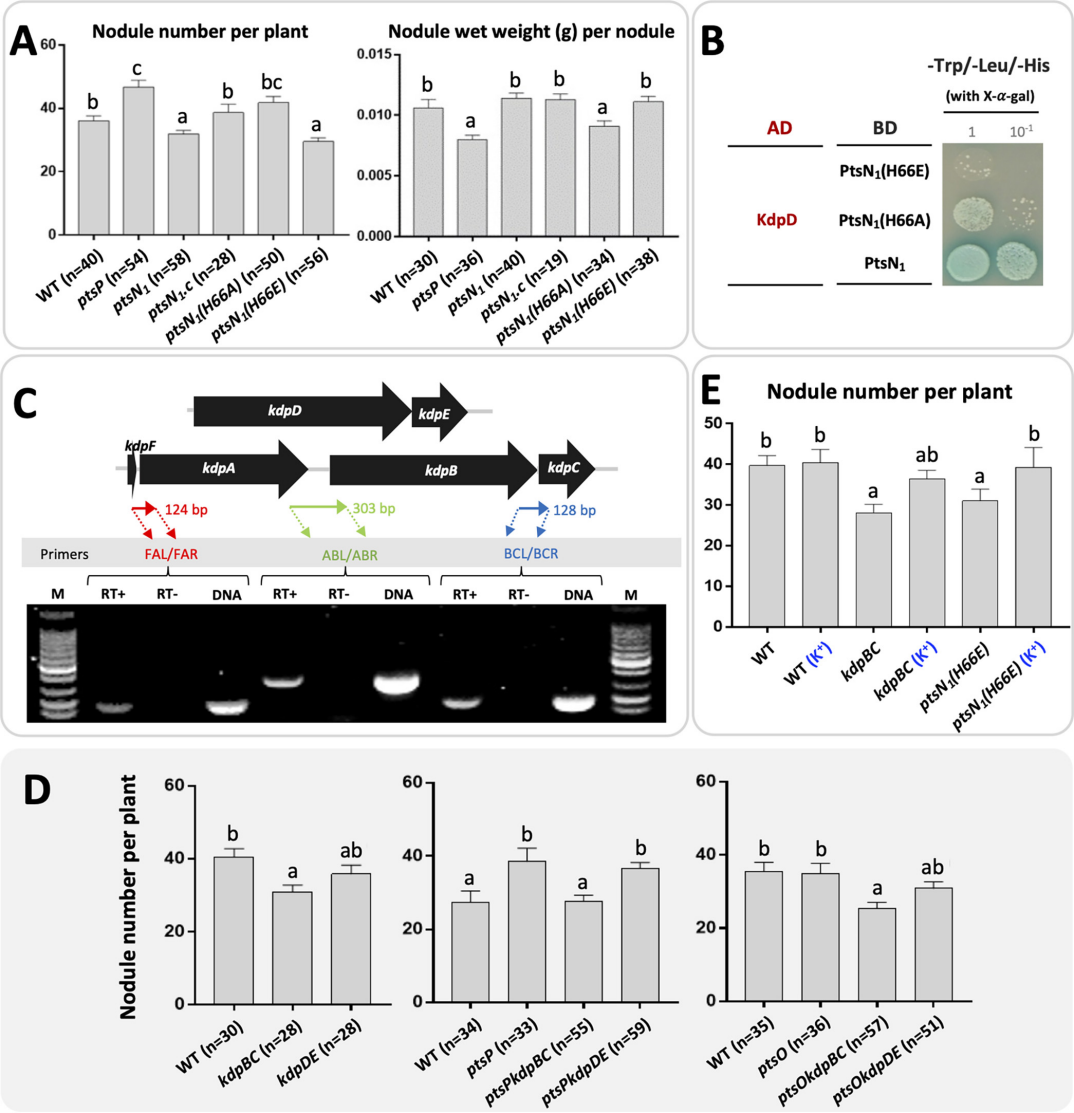
PTS^Ntr^ and KdpBC are required for optimal nodulation. (A) Nodulation characteristics of strains carrying *ptsN_1_(H66A)* or *ptsN_1_(H66E).* The number of scored plants from multiple independent experiments is shown in brackets. (B) PtsN_1_(H66A) directly interacts with KdpD. Two dilutions are shown from the yeast two-hybrid experiment with the negative (pGADT7 and pGBKT7) and positive (pGADT7-T and pGBKT7-53) controls as shown in Fig. 2 (C) Cotranscription of the *kdpFABC* operon of SF4 grown in minimum medium (M9). The fragments covering corresponding intergenic regions targeted by three pairs of primers are indicated and amplified in RT-PCR. Reverse transcriptase was added to the reaction in RT+, but omitted from reactions in RT-. Genomic DNA was amplified as a positive control. M, 100 bp marker. (D) Deletion of *kdpBC* rather than *kdpDE* in the wild-type SF4 (WT), the *ptsP* or *ptsO* mutants leads to less nodules formed on soybean plants. The number of scored plants is indicated in brackets. (E) Nodulation defects of the *kdpBC* mutant and the *ptsN_1_(H66E)* strain can be rescued by supplying replete K^+^ (10 mM) in the rhizosphere (more than 10 plants were scored). (A) and (D to E), different letters indicate significant difference between treatments (Average ± SEM; ANOVA followed by Duncan’s test, alpha = 0.05).

All known regulatory roles of EIIA^Ntr^ are mediated by its phosphorylation status of H66 (Fig. 1C) (8, 33, 40–42). Here we constructed the *ptsN_1_(H66A)* and *ptsN_1_(H66E)* strains harboring non-phosphorylated PtsN_1_ and phosphorylated PtsN_1_, respectively. Similar to the *ptsN_1_* mutant, the *ptsN_1_(H66E)* strain formed less nodules than the wild-type SF4, the *ptsP* mutant, and the *ptsN_1_(H66A)* strain while showing no significant difference in symbiotic performance regarding leaf chlorophyl content and shoot dry weight compared with these strains except the inefficient *ptsP* mutant (Fig. 4A; Table S3). Moreover, the *ptsN1(H66A)* strain and the *ptsP* mutant induced smaller nodules than the *ptsN_1_(H66E)* strain, the *ptsN_1_* mutant, and SF4 (Fig. 4A; Table S3). Therefore, the involvement of PtsN_1_ in optimal nodulation is mediated by the phosphorylation status of its H66 residue.

10.1128/mbio.03721-21.3 TABLE S3 Symbiotic performance of the *ptsN_1_(H66A)* and *ptsN_1_(H66E)* mutants on soybean plants. Download Table S3, PDF file, 0.1 MB. Copyright © 2022 Feng et al. 2022 Feng et al. https://creativecommons.org/licenses/by/4.0/ This content is distributed under the terms of the Creative Commons Attribution 4.0 International license.

Because the major EIIA^Ntr^ PtsN_1_ directly interacts with KdpD (Fig. 2), we wonder if the KdpDE-KdpFABC pathway is involved in optimal nodulation mediated by phosphorylation status of PtsN_1_. Yeast two-hybrid experiment showed that PtsN_1_(H66E) failed to interact with KdpD while PtsN_1_(H66A) can interact with KdpD though at a relatively lower efficiency compared with the wild-type PtsN_1_ (Fig. 4B). The interaction between unphosphorylated EIIA^Ntr^ and KdpD is consistent with the findings in other bacteria including E. coli and R. leguminosarum (15, 16).

The *kdpDE* and *kdpFABC* operons have a widely conserved synteny in various bacteria (43). In SF4 genome, the *kdpD* gene has four overlapping nucleotides with the downstream coding region of the response regulator KdpE (Fig. 4C), and reverse transcription-PCR analysis revealed that *kdpF*, *kdpA*, *kdpB*, and *kdpC*, encoding the high-affinity K^+^ uptake system, constitute an operon (Fig. 4C). To test the potential role of KdpDE and KdpFABC in nodulation, *kdpDE* and *kdpBC* were deleted in backgrounds of WT, *ptsP*, or *ptsO* mutants. All mutants lacking *kdpBC* formed less nodules compared with their parent strains whereas the decrease of nodule number for mutants lacking *kdpDE* was not statistically significant (Fig. 4D; Table S4), suggesting the requirement of high-affinity K^+^ uptake in optimal nodulation and potential complementary effects by other K^+^ uptake systems in the *kdpDE* mutant (see below for transcriptional profiles of different K^+^ uptake systems). The *kdp* mutants had similar symbiotic performance as their parent strains regarding leaf chlorophyll content and shoot dry weight (Table S4). When replete K^+^ (10 mM) was supplied in the rhizosphere, nodulation defects of the *kdpBC* and *ptsN_1_(H66E)* strains can be largely rescued (Fig. 4E). These results suggest that EIIA^Ntr^ and its downstream high-affinity K^+^ uptake system are involved in optimal nodulation on soybean plants. The involvement of K^+^ uptake system in modulating nodulation is also observed for S. meliloti associated with alfalfa (24) where the double mutant of low affinity K^+^ uptake systems Trk and Kup (14) exhibited delayed nodulation that was further enhanced in the *trk-kup-kdp* triple mutant. In line with the findings in this work on *S. fredii*-soybean system, these S. meliloti mutants of K^+^ uptake systems formed nitrogen fixing nodules on alfalfa (24), supporting the role of K^+^ uptake during early symbiotic interactions.

10.1128/mbio.03721-21.4 TABLE S4 Symbiotic performance of the *kdp* mutants on soybean plants. Download Table S4, PDF file, 0.1 MB. Copyright © 2022 Feng et al. 2022 Feng et al. https://creativecommons.org/licenses/by/4.0/ This content is distributed under the terms of the Creative Commons Attribution 4.0 International license.

**FIG 5:**
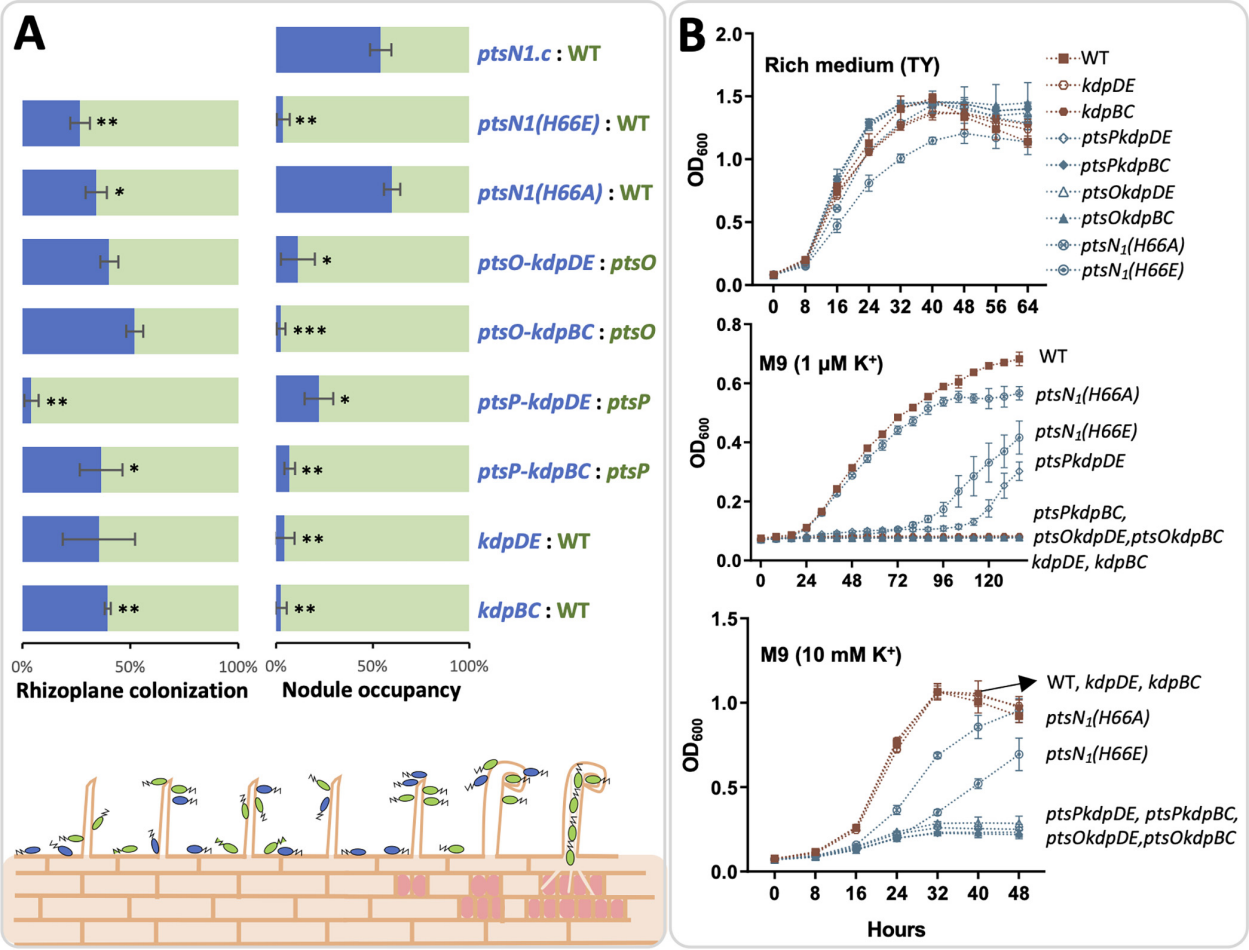
PTS^Ntr^ and Kdp system are required for nodule occupancy and low potassium adaptation. (A) Rhizoplane colonization and nodule occupancy by pairs of mixed inoculants (1:1 ratio within each pair). Significant difference is indicated based on one sample *t* test (theoretical mean = 0.5; *, *P* < 0.05; **, *P* < 0.01; ***, *P* < 0.001). Error bars represent SD of three biological replicates. (B) The growth curves of various derivatives of *S. fredii* CCBAU45436 (SF4) in the TY rich medium, M9 minimum medium with 1 μM or 10 mM K^+^.

In the S. meliloti-alfalfa system, it has been shown that the low affinity Trk and Kup systems are required for competitive nodulation (24). In this work, nodule occupancy assay on soybean plants (Fig. 5A) revealed that *S. fredii* mutants lacking *kdpBC* or *kdpDE* were outcompeted by their corresponding parent strains (WT, the *ptsP*, or *ptsO* mutants) while the *ptsN_1_(H66A)* strain rather than *ptsN_1_(H66E)* was as competitive as the wild-type SF4. Further analysis of survival rate (CFU) on rhizoplane showed that the observed contrasting competitive nodulation abilities among test strains (Fig. 4A; Fig. 4D; Fig. 5A) cannot be fully explained by their rhizoplane colonization rates (Fig. 5A). For example, rhizoplane CFU of the *kdpDE*, *ptsO-kdpDE*, and *ptsO-kdpBC* mutants were comparable with those of the corresponding parent strains. These findings imply a more active role of EIIA^Ntr^-Kdp pathway during symbiotic interactions than in root colonization.

To verify if the test mutants with nodulation defects are also impaired in low K^+^ adaptation, their growth curves were compared (Fig. 5B). Consistent with the predicted role of KdpDE and KdpFABC in low K^+^ adaptation (< 100 μM) (14, 44), the *kdpDE* and *kdpBC* mutants were unable to grow in the minimum medium containing 1 μM K^+^ while indistinguishable from the wild-type SF4 when replete K^+^ (10 mM) was supplied (Fig. 5B). Similarly, the *ptsP* or *ptsO* derivatives lacking either *kdpBC* or *kdpDE* showed significant growth defects under the low K^+^ condition (Fig. 5B), which can be rescued to the level of the *ptsP* and *ptsO* mutants (Fig. 3A) by supplying replete K^+^ (Fig. 5B). Under the low K^+^ condition, the *ptsN_1_(H66A)* strain grew at a similar rate as SF4 before reaching stationary phase while the *ptsN_1_(H66E)* strain showed a significant growth delay (Fig. 5B) that can be rescued to the level of the *ptsN_1_* mutant (Fig. 3A) by adding 10 mM K^+^ (Fig. 5B). It is also noteworthy that the unphosphorylated PtsN_1_ allowed better growth than the *ptsP* and *ptsO* mutants under the low K^+^ condition (Fig. 4B and Fig. 3A). This can be partially explained by EIIA^Ntr^-independent output signals derived from EI^Ntr^ and Npr as indicated in the carbon source utilization characteristics of related mutants of SF4 (29) and potential cross talk between the canonical PTS and PTS^Ntr^ in modulating the KdpDE-KdpFABC pathway as shown in E. coli (15, 45). Despite the complexity in the upstream signaling components, the unphosphorylated form of PtsN_1_ is notably essential for low K^+^ adaptation through interacting with KdpD (Fig. 5B and Fig. 4B).

### KdpD interacts with KdpE and PtsN_1_ in a non-canonical way.

**FIG 6:**
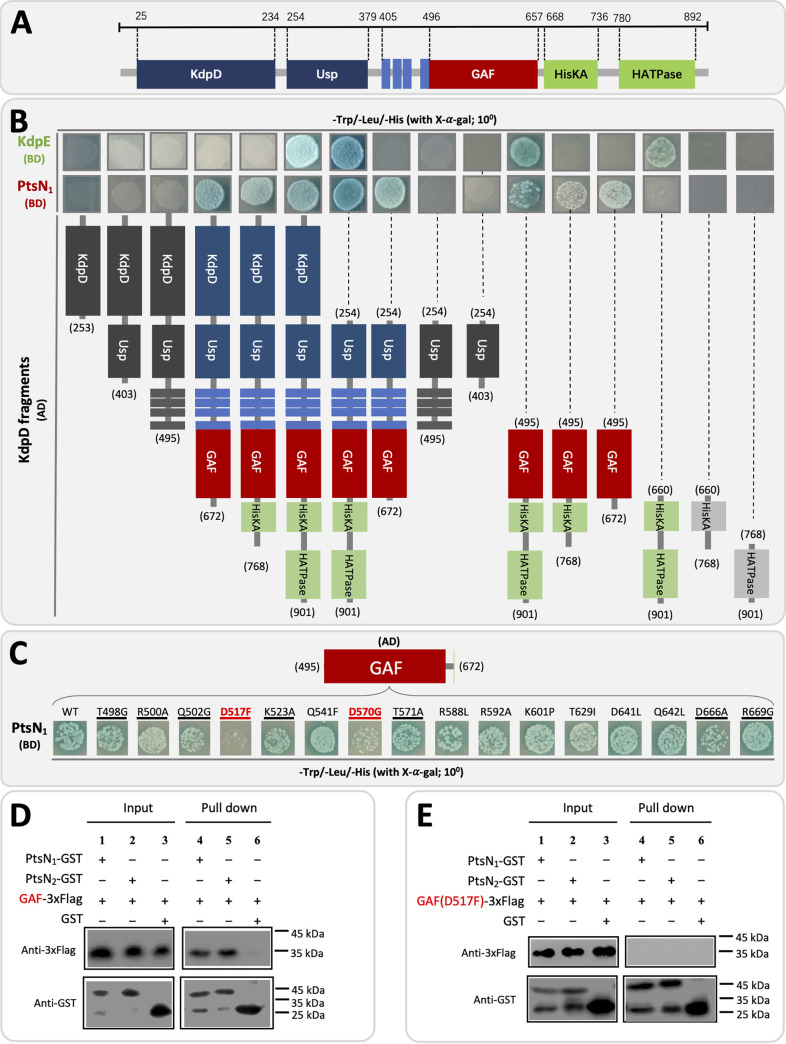
KdpD interacts with KdpE and PtsN_1_ by HisKA-HATPase and GAF, respectively. (A) Schematic view of KdpD domains. Four transmembrane domains are indicated in light blue. (B) Identification of KdpD fragments (AD) interacting with KdpE (BD) and PtsN_1_ (BD), respectively by using the yeast two-hybrid experiment. (C) Exploring screen of polar residues in the GAF domain involved in interacting with PtsN_1_. Amino acid substitutions are indicated and residues located in either N-terminal or C-terminal fragments which are present in various rhizobia but absent in E. coli are underlined. In the yeast two-hybrid experiment (B to C), pGADT7/pGBKT7 and pGADT7-T/pGBKT7-53 were used as negative and positive controls, respectively, as shown in Fig. 2. The relative position of domains and residues within KdpD are shown when necessary. (D) Interaction between PtsN_1_ or PtsN_2_ with the GAF domain of KdpD by using GST pulldown assay. (E) GAF(D517F) unable to interact with PtsN_1_ or PtsN_2_ in the GST pulldown assay.

The interaction between KdpD and PtsN_1_ has been demonstrated as mentioned above (Fig. 2 and Fig. 4B), though notable sequence variation was observed between PtsN homologs of rhizobia and E. coli (Fig. 1B and C). Further protein interaction analysis revealed the GAF domain as the minimum KdpD fragment interacting with PtsN_1_ while the minimum region covering HisKA and HATPase domains interacting with KdpE (Fig. 6A and B). In E. coli, the region covering HisKA and HATPase domains interacts with KdpE, but PtsN interacts with HisKA (44, 46), i.e., apparently competing for binding (43). This paradox is largely resolved in E. coli by forming the PtsN/KdpD_2_/KdpE ternary complex (46). Sequence analysis revealed that GAF of KdpD from SF4 and other rhizobia has additional N-terminal (from N496 to G529) and C-terminal (V650 to L672) fragments and more scattered polar residues (Q541, D570, T571, R588, R592, K601, T629, D641, and Q642) compared with GAF from E. coli (Fig. S2). Various GAF variants carrying substitutions at individual polar residues were constructed and tested for their interaction activity with PtsN_1_ (Fig. 6C). It turned out that D517 located in the N-terminal fragment and D570, not present in GAF of E. coli KdpD, were the key residues involved in the interaction between GAF of KdpD and PtsN_1_ in SF4. This novel interaction mechanism between KdpD and EIIA^Ntr^ was further confirmed in the GST pulldown assay where intact GAF of KdpD rather than GAF(D517F) can effectively interact with PtsN_1_ or PtsN_2_ (Fig. 6D and E). Because the D517 carrying N-terminal fragment is also present in KdpD of many other rhizobia (Fig. S2), this signal transduction mechanism represents a novel model alternative to the well-known PtsN/KdpD_2_/KdpE ternary binding model based on findings in E. coli (43, 46).

10.1128/mbio.03721-21.8 FIG S2 Alignment of GAF domain of KdpD from representative rhizobia and E. coli. Polar residues subject to point mutation in Fig. 6D are indicated. The green box shows regions absent in GAF of KdpD from E. coli. Identity levels are indicated in navy blue (100%), pink (75%), and azure (50%~75%). Download FIG S2, PDF file, 0.5 MB. Copyright © 2022 Feng et al. 2022 Feng et al. https://creativecommons.org/licenses/by/4.0/ This content is distributed under the terms of the Creative Commons Attribution 4.0 International license.

### KdpE directly binds the promoter of *kdpFABC* but not those of *trkA* and *kup*.

**FIG 7:**
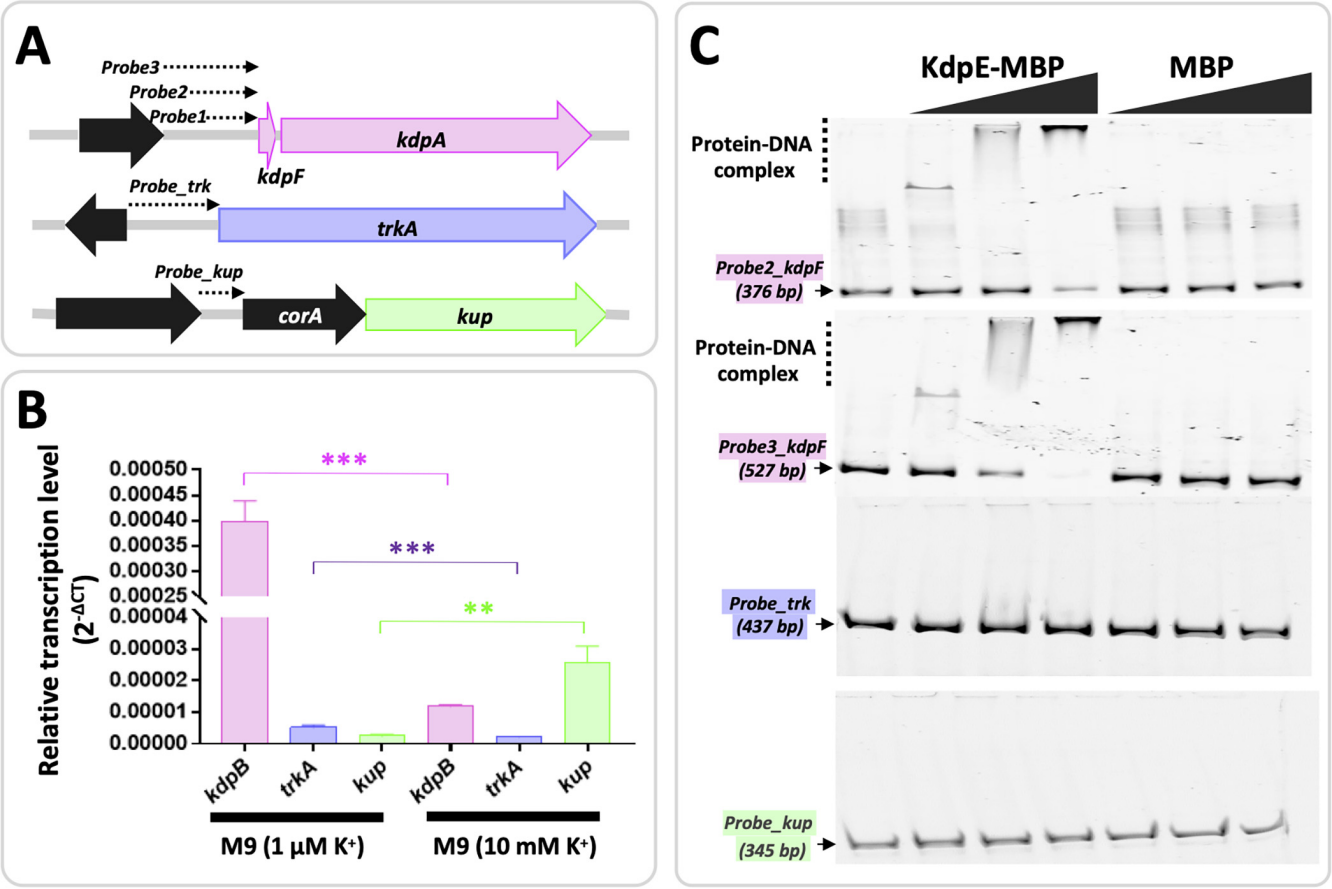
KdpE binds promoter of *kdpFABC* but not those of *trkA* and *kup*. (A) Promoters of *kdpFABC*, *trkA* and *kup*. The positions of probes used in electrophoretic mobility shift assay (EMSA) are indicated. *corA* encodes a putative transport protein for magnesium and cobalt. (B) qRT-PCR analysis of *kdpB*, *trkA*, and *kup* genes in SF4 under 1 μM and 10 mM K^+^ conditions in the M9 minimum medium. 16S rRNA gene is used as the reference gene. (C) EMSA of KdpE with *kdpFABC*/*trkA*/*kup* promoter regions. The purified KdpE-MBP and MBP (5/20/50 μM) were incubated with Cy5-labeled DNA probes (12.3 nM). KdpE-MBP did not bind the *Probe1_kdpF* and the result is not shown herein.

In addition to the high-affinity K^+^ uptake system KdpFABC, the genome of SF4 harbors homologs of low-affinity TrkA and Kup systems (Fig. 7A) (24, 47, 48). qRT-PCR analysis of SF4 revealed that *kup* was downregulated while *kdp* was strongly upregulated under the low K^+^ condition compared to the replete K^+^ condition (Fig. 7B). By contrast, the *trkA* gene was transcribed at a relatively lower level compared with the other two systems when replete K^+^ was supplied in the minimum medium, and it was slightly upregulated in the low K^+^ medium (Fig. 7B). Further electrophoretic mobility shift assay (EMSA) showed that KdpE can directly binds the promoter region of *kdpFABC* operon but not those of *trkA* and *kup* (Fig. 7C). Therefore, the direct activation of *kdpFABC* operon by KdpE (43) also function in *S. fredii*.

### The role of EIIA^Ntr^-KdpDE-KdpFABC pathway during early symbiotic interactions.

**FIG 8:**
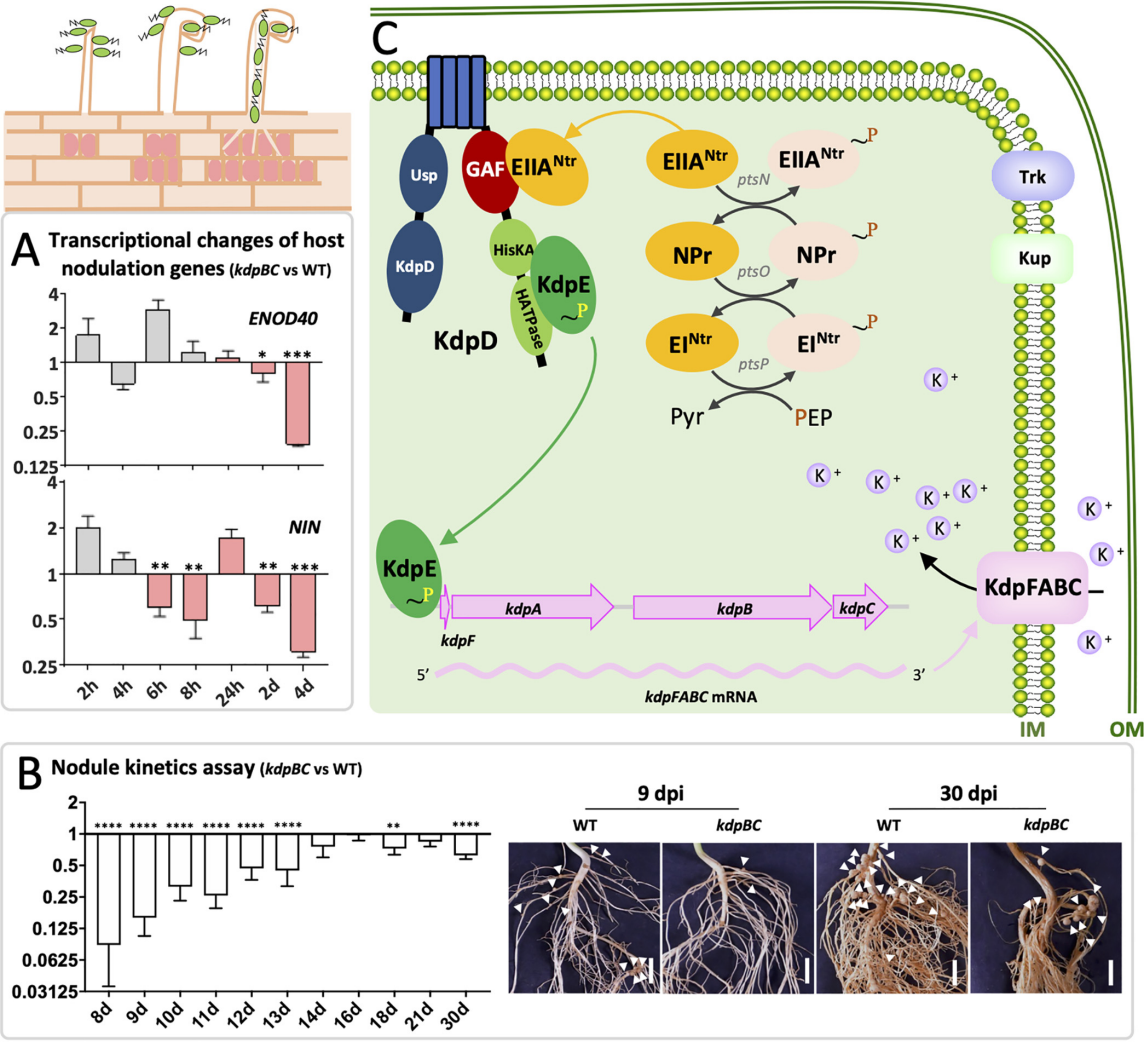
Regulation of K^+^ uptake by the PTS^Ntr^-KdpDE-KdpFABC pathway in nodulation. (A) Down regulation of *NIN* and *ENOD40* in roots inoculated with the *kdpBC* mutant compared with those in roots treated with the wild-type SF4. Hours (h) or days (d) postinoculation are shown corresponding to the early symbiotic interaction stages. The red bars represent a significant upregulation in the WT treatment compared with the uninoculated roots. Significant differences in gene transcriptional levels between the *kdpBC* treatment and the WT treatment are indicated (Student’s *t* test; *, *P* < 0.05; **, *P* < 0.01; ***, *P* < 0.001). Error bars represent SE of the mean of three biological replicates. (B) Delayed nodulation of the *kdpBC* mutant compared with SF4 (dpi, days postinoculation). Significant differences in nodule number between two treatments are indicated (Student’s *t* test; **, *P* < 0.01; ****, *P* < 0.0001). Error bars represent SE (based on data from two independent experiments; 6 to 11 plants were scored at each time point in each independent experiment). White triangles indicate position of nodules. Scale bars, 1 cm. (C) During nodulation, unphosphorylated form of EIIA^Ntr^ interacts with the GAF domain of KdpD, which activates KdpE through the direct interaction between KdpE and the HisKA-HATPase region of KdpD. The active KdpE in turn binds the promoter of the *kdpFABC* operon to upregulate the transcription of this high-affinity K^+^ uptake system.

The above-mentioned direct evidences support a EIIA^Ntr^-KdpDE-KdpFABC pathway in *S. fredii*, mediated by a non-canonical EIIA^Ntr^-KdpD-KdpE binding model. It is noteworthy that SF4 derivatives carrying the phosphorylated form of PtsN_1_(H66E) or lacking *kdpBC* formed less nodules on soybean plants while the number of nodules induced by the *kdpDE* mutant was not significantly different from that of the wild-type SF4 (Fig. 4A and D). Transcriptional profiles of *kdp*, *trkA*, and *kup* genes were determined under both low and replete K^+^ conditions (Fig. S3). The deletion of *kdpDE* led to low transcription of the high-affinity KdpFABC system compared with SF4 as expected (Fig. S3), whereas the *kup* gene was strongly upregulated under the low K^+^ condition (Fig. S3A; around 50-fold increase compared with SF4) though downregulated when 10 mM K^+^ was supplied (Fig. S3B). For those strains forming less nodules such as the *ptsN_1_(H66E)* and *kdpBC* strains, the *kup* and/or *trkA* were downregulated under the low K^+^ condition (Fig. S3). By contrast, the *ptsN_1_(H66A)* strain forming more nodules had a significant higher transcriptional level of *kdp* under both low and replete K^+^ conditions, though *trkA* and *kup* were downregulated under the low K^+^ condition (Fig. S3). The cumulative contribution of different K^+^ uptake systems to nodulation was also observed in the S. meliloti-alfalfa symbiosis (24). In this work, we further revealed that the optimal nodulation in the *S. fredii*-soybean system is modulated by the EIIA^Ntr^-KdpDE-KdpFABC pathway. Further exploring host responses to the *kdpBC* mutant during early symbiotic interaction stages (2-h, 4-h, 6-h, 8-h, 24-h, 2-days, and 4-days postinoculation) revealed an impaired transcription of the indispensable nodule inception regulator gene *NIN* and nodule primordium initiation marker gene *ENOD40* (28, 49–51) in soybean roots (Fig. 8A). This is consistent with the significantly delayed nodulation of the *kdpBC* mutant compared with the wild-type SF4 (Fig. 8B). Taken together with the findings on rhizoplane colonization and nodule occupancy, it seems that rhizobial K^+^ uptake modulated by the PTS^Ntr^-KdpDE-KdpFABC is crucial during early symbiotic interactions (Fig. 8C).

10.1128/mbio.03721-21.9 FIG S3 Transcription profiles of potassium uptake systems in related mutants of the EIIA^Ntr^-KdpDE-KdpFABC pathway. (A, B) qRT-PCR analysis of *kdpB*, *trkA*, and *kup* genes in the *ptsN1(H66A)*, *ptsN1(H66E), kdpDE*, and *kdpBC* mutants under 1 μM (A) and 10 mM (B) K^+^ conditions in the M9 minimum medium. The reference gene is 16S rRNA gene. Significant difference is indicated based on one sample t test, theoretical mean = 1; red and blue represent significant up- and down-regulation, respectively, at *P < *0.05 or at the marginal *P* values as indicated (three biological replicates with three technical replicates). Download FIG S3, PDF file, 0.2 MB. Copyright © 2022 Feng et al. 2022 Feng et al. https://creativecommons.org/licenses/by/4.0/ This content is distributed under the terms of the Creative Commons Attribution 4.0 International license.

### Conclusion.

Despite the expansion of PtsN homologs in *S. fredii*, PtsN_1_ is the major EIIA^Ntr^ functioning in low K^+^ adaptation and optimal nodulation, which are mediated by the two-component system KdpDE and the high-affinity K^+^ uptake system KdpFABC. The sensor kinase KdpD interacts with the unphosphorylated form of EIIA^Ntr^ in a novel mechanism via its GAF domain, and with the response regulator KdpE via the HisKA-HATPase fragment. KdpE directly activates the transcription of the *kdpFABC* operon. Disruption of this pathway leads to defects in low K^+^ adaptation and competitive nodulation. The *kdpBC* mutant has a reduced nodulation ability compared with WT while showing no severe impairment in rhizoplane colonization. This can be at least partially explained by the impaired induction of host nodulation genes by the *kdpBC* mutant and its delayed nodulation. Collectively, these findings suggest that K^+^ uptake regulated by the PTS^Ntr^-KdpDE-KdpFABC pathway is involved in optimizing early symbiotic interactions, highlighting a largely unexplored regulation of symbiosis by fluctuating nutrients in soils (31). K^+^ is needed by all cellular organisms (43) and its role as an environmental cue in bacteria-host interactions is just emerging (26).

## MATERIALS AND METHODS

### Strains, plasmids, primers, and growth conditions.

Strains and plasmids used in this study are listed in Table S5. All primers are shown in Table S6. *S. fredii* strains were grown at 28°C in Tryptone-Yeast (TY) (52) or modified-M9 minimal medium (53), with 1 μM, 1 mM, 5 mM, 10 mM, or 20 mM KCl supplied as indicated. E. coli was grown at 37°C in Luria-Bertani (LB) medium. Saccharomyces cerevisiae was grown at 30°C in Yeast-Peptone-Dextrose (YPD) medium (yeast extract 10 g/L, peptone 20 g/ L, glucose 20 g/ L). Antibiotics were added when necessary as described previously (29, 31). The Bioscreen C (Oy Growth Curves Ab Ltd, Raisio, Finland) was used to determine growth curves of test strains.

10.1128/mbio.03721-21.5 TABLE S5 Strains and plasmids used in this work. Download Table S5, XLSX file, 0.02 MB. Copyright © 2022 Feng et al. 2022 Feng et al. https://creativecommons.org/licenses/by/4.0/ This content is distributed under the terms of the Creative Commons Attribution 4.0 International license.

10.1128/mbio.03721-21.6 TABLE S6 Primers used in this work. Download Table S6, XLSX file, 0.02 MB. Copyright © 2022 Feng et al. 2022 Feng et al. https://creativecommons.org/licenses/by/4.0/ This content is distributed under the terms of the Creative Commons Attribution 4.0 International license.

### Plant assays, competitive nodulation, and rhizoplane colonization.

Seeds of soybean cultivar JD17 (54) were treated with 95% ethanol for 30 s, then surface sterilized in 17% (vol/vol) NaClO for 3 min, and washed five to seven times using autoclaved deionized water. These seeds were germinated on 0.5% agar plates at 28°C in the dark for 48 h. Seedlings were inoculated with 1 mL of rhizobial suspension with OD_600_ = 0.2 in 0.8% (wt/vol) NaCl solution, and cultivated in vermiculite moistened with low-N nutrient solution [Ca(NO_3_)_2_·4H_2_O 0.03 g, KCl 0.075 g, MgSO_4_ 0.06 g, K_2_HPO_4_ 0.136 g, CaSO_4_·2H_2_O 0.46 g, FeC_6_H_5_O_7_ 0.075 g, H_3_BO_3_ 2.86 mg, MnSO_4_ 1.81 mg, CuSO_4_·5H_2_O 0.8 mg, ZnSO_4_ 0.22 mg, H_2_MoO_4_ 0.02 mg in one L medium]. When necessary, 10 mM KCl was added into the low-N nutrient solution. Plants were harvested 30 days postinoculation or as indicated for nodule kinetics assay. The leaf chlorophyll content and shoot dry weight were determined as described previously (29). To determine nodule occupancy of rhizobia, the mutants were mixed with their parent strains at 1:1 (OD_600_ = 0.2) and inoculated on soybean plants. At 30 dpi, nodules were surface sterilized and nodulating strains were identified by their growth on TY plates with or without corresponding antibiotics as described previously (29). The identity of strains was further verified by PCR using primers targeting strain-specific fragments. The rhizoplane colonization ability was determined using the procedure described previously (31). Briefly, five to six germinated seeds were transferred to sterile petri dishes (diameter, 9 cm) which have low-N nutrient medium with 0.8% agar and filter paper. The mutants were mixed with their parent strains in equal quantity and inoculated (OD_600_=0.2) on seedlings. At 7 dpi, roots were washed with water for three times and suspended in 0.85% NaCl solution. After exposure to six cycles of 30-s ultrasound treatment, the suspension was diluted and plated on TY plates with corresponding antibiotics. Colonies were counted and used for PCR verification of bacterial identity.

### In-frame deletion and point mutation in *S. fredii*.

In-frame deletion of *ptsN* homologs and *kdp* genes were performed using the seamless assembly cloning kit (Taihe Biotechnology, Beijing, China) with various pJQ200SK (55) derivatives carrying corresponding upstream and downstream homologous fragments using the procedure described previously (36). Upstream and downstream fragments were obtained by PCR using primers carrying sequences corresponding to the ends of SpeI restriction sites in pJQ200SK. The resultant homologous fragments were mixed with the linearized pJQ200SK (digested by SepI) in the reaction buffer that was further incubated at 50°C for 15 min before the transformation experiment with E. coli DH5α. The correct engineered plasmids harbored by positive clones were verified using PCR and Sanger sequencing, and then conjugated into *S. fredii* strains with the helper plasmid pRK2013 (56). Single-crossover clones resistant to gentamicin were further subject to counterselection for double recombinants using 5% sucrose. Double-crossover clones were verified by colony PCR and Sanger sequencing.

The *ptsN_1_(H66A)*, *ptsN_1_(H66E)*, and *ptsN_1_.c* strains were constructed using the seamless assembly cloning kit (Taihe Biotechnology, Beijing, China) with pVO155 (57) derivatives carrying the wild-type *ptsN_1_* sequence (*ptsN1.c*) or its mutated forms (H66A: CAC to TGC; H66E: CAC to TTC). The pVO155 was linearized by BamHI and XbaI, and mixed with the corresponding *ptsN_1_* fragments in the reaction buffer, and incubated at 50°C for 15 min. The resultant plasmids were transformed into E. coli DH5α and positive clones were verified by PCR and Sanger sequencing. The plasmids were then conjugated into the *ptsN_1_* mutant with the helper plasmid pRK2013 and single-crossover clones resistant to kanamycin were verified by colony PCR and Sanger sequencing.

### Yeast two-hybrid assay.

By using the seamless assembly cloning method as described above, PtsN_1_, PtsN_2_, PtsN_3_, KdpE, KdpD, different fragments of KdpD, or mutated GAF of KdpD were fused to either the GAL4 activation domain (AD; pGADT7) or DNA-binding domain (BD; pGBKT7) to generate various derivatives which were then transformed into E. coli DH5α and positive clones were verified by PCR and Sanger sequencing. The extracted various AD and BD plasmids were cointroduced into Saccharomyces cerevisiae AH109. The yeast two-hybrid assay was performed according to the manual of Matchmaker GAL4 two-hybrid system 3 (TaKaRa Bio).

### Protein purification, pulldown assay, and Western blot analysis.

Sequences corresponding to GAF of KdpD, GAF(D517F), PtsN_1_, PtsN_2_, and KdpE were amplified using related primers as shown in Table S6. The resultant fragments were ligated into expression vector pET-28a(+), pGEX-4T-1, or pMAL-c5x, and transformed into E. coli BL21(DE3), generating the GAF-3×Flag, GAF(D517F)-3×Flag, PtsN_1_-GST, PtsN_2_-GST, and KdpE-MBP expression vectors. Cultures of OD_600_ = 0.4 to 0.6 were subject to induction with 0.1 mM IPTG (Coolaber) for 14 h at 16°C. Cells were then harvested, washed, and resuspended using lysis buffer with protease inhibitor cocktail (Coolaber), and sonicated on ice. GAF-3×Flag and GAF(D517F)-3×Flag cell extracts were loaded onto Nickel-IDA agarose beads (GenStar), and KdpE-MBP cell extracts were loaded onto Amylose Resin (BioLabs) for protein purification.

To perform GST pulldown assay, PtsN_1_-GST and PtsN_2_-GST cell extracts were incubated with GST beads for 3 h at 4°C, and washed three times with low salt buffer (20 mM Tris-HCl [pH 7.5], 150 mM NaCl, 0.1% Triton X-100, and protease inhibitor cocktail [Coolaber]). Then the beads were incubated in blocking buffer (20 mM Tris-HCl [pH 7.5], 150 mM NaCl, 0.1% Triton X-100, 5% BSA, protease inhibitor cocktail) for 1h at 4°C. The beads were incubated with GAF-3×Flag or GAF(D517F)-3×Flag proteins at 4°C for 1h. Finally, the beads were collected and washed three to five times with low salt buffer and high salt buffer (20 mM Tris-HCl [pH 7.5], 300 mM NaCl, 0.1% Triton X-100, protease inhibitor cocktail). The proteins eluted from beads were then used for Western bolt analysis.

Proteins were added into 5× loading buffer, then boiled 5 min, and electrophoresed on 10% SDS-PAGE gels. Monoclonal mouse antibody against 3×Flag epitope (Sigma) or GST (Sigma) epitope and the horseradish peroxidase (HPR)-conjugated goat anti-mouse immunoglobulin G (IgG) secondary antibody (ZSGB-BIO) were used at 1:500 and 1:1,000 dilution ratios. Signals of the protein on X-ray film were recorded by chemiluminescence detection.

### Electrophoretic mobility shift assay.

The Cy5-DNA probes (*Probe1_kdpF*, *Probe2_kdpF*, *Probe3_kdpF*, *Probe_trk*, and *Probe_kup*) within the putative promoter regions of *kdpFABC*, *trkA*, or *kup* were amplified with related primers labeled with Cy5 at 5′ ends (Table S6). Different quantity of purified KdpE-MBP or MBP (5, 20, 50 μM) and 12.3 nM individual Cy5-DNA probes were added into the 10 μL reaction mixture (0.5 mg/mL BSA, 0.1 mg/mL sonicated salmon sperm DNA, 25 mM Tris-HCl [pH 8.0], 5% glycerol, 0.05% DDM) and incubated at 20°C for 30 min. The resultant samples were separated in a 6% (wt/vol) native polyacrylamide gel and visualized with a Typhoon FLA 9000 imager (GE Healthcare).

### The *kdpFABC* operon determination and quantitative real-time PCR.

To determine the cotranscription of *kdpF*, *kdpA*, *kdpB*, and *kdpC*, reverse transcription-PCR was conducted. The concentration of mid-log-phase cultures was adjusted to OD_600_ = 0.2 and cultured in modified M9 minimal medium for 9 h. RNA was extracted using a Bacteria Total RNA Kit (Zomanbio). cDNA was synthesized using Reverse Transcriptase Kit (Zomanbio). Primers targeting for intergenic regions of *kdpF-kdpA*, *kdpA-kdpB*, or *kdpB-kdpC* were used to test the cotranscription profiles in cDNA, with DNA as control samples.

To determine the transcriptional profiles of *kdpB*, *trkA*, and *kup* in various test strains, rhizobia were cultivated in modified M9 minimum medium (supplied with 1 μM or 10 mM KCl) for 9 h as described above. Extraction of rhizobial RNA and cDNA synthesis were carried out using the same method described above. To test the transcriptional levels of *NIN* and *ENOD40* in soybean roots during early symbiotic interactions, roots from soybean plants inoculated with the *kdpBC* mutant or the wild-type SF4 were collected at 2-h, 4-h, 6-h, 8-h, 24-h, 2-days and 4-days postinoculation, and the uninoculated roots at the same stages were used as control. The Total RNA Extraction Kit (Promega) and Reverse Transcriptase Kit (Genestar) were used to obtain root RNA and cDNA. The qRT-PCR was performed with corresponding gene-specific primers using RealStar Green Fast Mixture (Genestar) and an ABI QuantStudioT^6^ Flex System real-time PCR system. Transcription levels were normalized to the expression of the internal control gene 16s rRNA (bacteria) or 18s rRNA (plants). Three biological replicates were performed.

### Phylogenetic analysis.

Protein sequences of PtsN homologs were extracted from the GenBank database, aligned with ClustalW, and used in the maximum likelihood phylogenetic tree reconstruction by MEGA5 (58) with default parameters. The tree was tested by 1,000 bootstrap replicates.
