# Supplementary material for: The PTSNtr-KdpDE-KdpFABC Pathway Contributes to Low Potassium Stress Adaptation and Competitive Nodulation of Sinorhizobium fredii
Source: mBio. 2022 May 2;13(3):e03721-21. doi: 10.1128/mbio.03721-21 (PMC9239096; doi:10.1128/mbio.03721-21)
Supplement: TABLE S1 [file mbio.03721-21-s0004.pdf]

Table S1 Symbiotic performance of *ptsN* mutants on soybean plants.

Treatment	Chlorophyll content (SPAD value)	Shoot dry weight (g/plant)	Nodule number (per plant)	Nodule wet weight (g/plant)
WT	35.6 ± 1.6 (bc)	0.55± 0.03 (bcd)	39.1 ± 1.9 (b)	0.25 ± 0.01 (abc)
ptsN1	35.3 ± 1.2 (b)	0.50± 0.03 (b)	26.1 ± 1.6 (a)	0.23 ± 0.01 (ab)
ptsN2	33.9 ± 1.2 (bc)	0.44± 0.03 (bc)	27.3 ± 1.3 (a)	0.22 ± 0.01 (a)
ptsN3	37.5 ± 1.0 (bc)	0.48± 0.03 (bc)	35.8 ± 1.7 (b)	0.25 ± 0.01 (abc)
ptsN12	34.2 ± 1.4 (bc)	0.49± 0.03 (b)	29.7 ± 1.7 (a)	0.23 ± 0.01 (ab)
ptsN13	34 ± 1.2 (bc)	0.47 ± 0.02 (bc)	25.1 ± 1.2 (a)	0.23 ± 0.01 (a)
ptsN23	34.2 ± 0.9 (c)	0.49 ± 0.02 (cd)	37.4 ± 1.3 (b)	0.28 ± 0.01 (c)
ptsN123	37.8 ± 0.7 (bc)	0.57 ± 0.03 (cd)	27.7 ± 1.9 (a)	0.27 ± 0.02 (bc)
Control	26 ± 1.2 (a)	0.33 ± 0.01 (a)		

Different letters in brackets indicate significant difference between treatments (Average ± SE; ANOVA followed by Duncan's test, alpha = 0.05). More than 30 plants were scored.
