# Supplementary material for: The PTSNtr-KdpDE-KdpFABC Pathway Contributes to Low Potassium Stress Adaptation and Competitive Nodulation of Sinorhizobium fredii
Source: mBio. 2022 May 2;13(3):e03721-21. doi: 10.1128/mbio.03721-21 (PMC9239096; doi:10.1128/mbio.03721-21)
Supplement: FIG S1 [file mbio.03721-21-s0001.pdf]

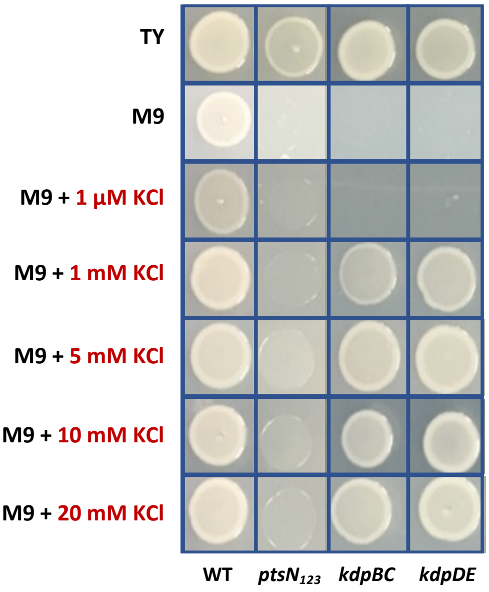

Fig. S1. Influence of K^+ levels on the growth of the *kdpBC*, *kdpDE* and *ptsN$_{123}$* mutants in the minimum medium.
