# Supplementary material for: The PTSNtr-KdpDE-KdpFABC Pathway Contributes to Low Potassium Stress Adaptation and Competitive Nodulation of Sinorhizobium fredii
Source: mBio. 2022 May 2;13(3):e03721-21. doi: 10.1128/mbio.03721-21 (PMC9239096; doi:10.1128/mbio.03721-21)
Supplement: TABLE S2 [file mbio.03721-21-s0005.pdf]

Table S2 Symbiotic performance of *ptsPN₁* and *ptsPN₁₂* mutants on soybean plants.

Treatment	Chlorophyll content (SPAD value)	Shoot dry weight (g/plant)	Nodule number (per plant)	Nodule wet weight (g/plant)	Nodule wet weight (g/nodule)
**WT**	36.9 ± 0.9 (c)	0.54 ± 0.06 (c)	37.8 ± 4.1 (bc)	0.32 ± 0.03 (b)	0.009 ± 0.001 (bc)
ptsP	16.0 ± 1.0 (a)	0.31 ± 0.04 (ab)	42.8 ± 3.4 (c)	0.19 ± 0.03 (a)	0.005 ± 0.001 (a)
ptsN₁	35.4 ± 1.1 (c)	0.57 ± 0.07 (c)	27.9 ± 2.0 (ab)	0.31 ± 0.03 (b)	0.011 ± 0.012 (c)
ptsPN₁	28.1 ± 1.5 (b)	0.43 ± 0.05 (bc)	35.4 ± 4.7 (bc)	0.29 ± 0.03 (b)	0.008 ± 0.001 (b)
ptsPN₁₂	25.3 ± 2.5 (b)	0.36 ± 0.05 (ab)	23.9 ± 2.7 (a)	0.18 ± 0.02 (a)	0.008 ± 0.001 (b)
**Control**	15.3 ± 1.2 (a)	0.27 ± 0.02 (a)			

The data of Table S1 and Table S2 are obtained simultaneously, *WT*, *ptsN₁* and control are used for both Table S1 and Table S2. Different letters in brackets indicate significant difference between treatments (Average ± SE; ANOVA followed by Duncan's test, alpha = 0.05). More than eight plants were scored.
