# Supplementary material for: The PTSNtr-KdpDE-KdpFABC Pathway Contributes to Low Potassium Stress Adaptation and Competitive Nodulation of Sinorhizobium fredii
Source: mBio. 2022 May 2;13(3):e03721-21. doi: 10.1128/mbio.03721-21 (PMC9239096; doi:10.1128/mbio.03721-21)
Supplement: TABLE S3 [file mbio.03721-21-s0006.pdf]

Table S3 Symbiotic performance of the *ptsN₁(H66A)* and *ptsN₁(H66E)* mutants on soybean plants.

Treatment	Chlorophyll content (SPAD value)	Shoot dry weight (g/plant)	Nodule number (per plant)	Nodule wet weight (g/plant)	Nodule wet weight (g/nodule)
**WT**	40.4 ± 0.8 (b)	0.85 ± 0.09 (b)	39.6 ± 1.9 (b)	0.38 ± 0.02 (bc)	0.011 ± 0.001 (b)
ptsP	18.1 ± 0.8 (a)	0.46 ± 0.05 (a)	46.7 ± 2.1 (c)	0.34 ± 0.02 (ab)	0.008 ± 0.001 (a)
ptsN₁	40.6 ± 0.9 (b)	0.79 ± 0.07 (b)	31.8 ± 1.2 (a)	0.36 ± 0.01 (abc)	0.011 ± 0.001 (b)
ptsN₁.c	40.4 ± 0.8 (b)	0.74 ± 0.06 (b)	38.7 ± 2.6 (b)	0.41 ± 0.02 (c)	0.011 ± 0.001 (b)
ptsN₁(H66A)	42.9 ± 0.8 (b)	0.75 ± 0.08 (b)	41.8 ± 1.9 (bc)	0.36 ± 0.02 (abc)	0.009 ± 0.001 (a)
ptsN₁(H66E)	40.6 ± 0.8 (b)	0.71 ± 0.07 (b)	29.5 ± 1.1 (a)	0.31 ± 0.01 (a)	0.011 ± 0.001 (b)
**Control**	18.9 ± 0.6 (a)	0.44 ± 0.04 (a)			

Different letters in brackets indicate significant difference between treatments (Average ± SE; ANOVA followed by Duncan's test, alpha = 0.05). More than 19 plants were scored.
