# Supplementary material for: The PTSNtr-KdpDE-KdpFABC Pathway Contributes to Low Potassium Stress Adaptation and Competitive Nodulation of Sinorhizobium fredii
Source: mBio. 2022 May 2;13(3):e03721-21. doi: 10.1128/mbio.03721-21 (PMC9239096; doi:10.1128/mbio.03721-21)
Supplement: TABLE S4 [file mbio.03721-21-s0007.pdf]

Table S4 Symbiotic performance of the *kdp* mutants on soybean plants.

Treatment	Chlorophyll content (SPAD value)	Shoot dry weight (g/plant)	Nodule number (per plant)	Nodule wet weight (g/plant)	Nodule wet weight (g/nodule)
**WT**	40.4 ± 0.8 (b)	0.85 ± 0.09 (b)	40.5 ± 2.4 (b)	0.38 ± 0.02 (a)	0.009 ± 0.001 (a)
kdpBC	41.5 ± 0.8 (b)	0.80 ± 0.08 (b)	31.0 ± 1.9 (a)	0.39 ± 0.02 (a)	0.013 ± 0.001 (b)
kdpDE	40.4 ± 1.1 (b)	0.85 ± 0.10 (b)	35.9 ± 2.4 (ab)	0.37 ± 0.02 (a)	0.010 ± 0.001 (a)
**Control**	21.1 ± 0.6 (a)	0.44 ± 0.04 (a)			
**WT**	39.7 ± 0.7 (b)	0.78 ± 0.07 (b)	27.4 ± 3.1 (a)	0.29 ± 0.03 (a)	0.010 ± 0.001 (c)
ptsP	23.9 ± 1.2 (a)	0.44 ± 0.04 (a)	38.5 ± 3.6 (b)	0.26 ± 0.03 (a)	0.007 ± 0.001 (a)
ptsPkdpBC	25.0 ± 0.8 (a)	0.45 ± 0.03 (a)	27.8 ± 1.6 (a)	0.22 ± 0.02 (a)	0.009 ± 0.001 (b)
ptsPkdpDE	24.4 ± 0.8 (a)	0.46 ± 0.02 (a)	36.6 ± 1.6 (b)	0.27 ± 0.02 (a)	0.008 ± 0.001 (ab)
**Control**	23.2 ± 0.9 (a)	0.43 ± 0.03 (a)			
**WT**	41.0 ± 0.8 (b)	0.78 ± 0.07 (b)	35.5 ± 2.6 (b)	0.38 ± 0.02 (b)	0.009 ± 0.001 (a)
ptsO	23.3 ± 0.8 (a)	0.45 ± 0.04 (a)	35.0 ± 2.7 (b)	0.31 ± 0.02 (a)	0.009 ± 0.001 (a)
ptsOkdpBC	22.8 ± 0.7 (a)	0.42 ± 0.02 (a)	25.4 ± 1.7 (a)	0.30 ± 0.01 (a)	0.010 ± 0.001 (a)
ptsOkdpDE	21.9 ± 0.6 (a)	0.44 ± 0.02 (a)	30.9 ± 1.8 (ab)	0.30 ± 0.02 (a)	0.009 ± 0.001 (a)
**Control**	21.1 ± 3.6 (a)	0.43 ± 0.03 (a)			

This table includes three sets of experiments distinguished by backgrounds. Within individual sets, different letters in brackets indicate significant difference between treatments (Average ± SE; ANOVA followed by Duncan's test, alpha = 0.05). More than 28 plants were scored.
