# Supplementary material for: The PTSNtr-KdpDE-KdpFABC Pathway Contributes to Low Potassium Stress Adaptation and Competitive Nodulation of Sinorhizobium fredii
Source: mBio. 2022 May 2;13(3):e03721-21. doi: 10.1128/mbio.03721-21 (PMC9239096; doi:10.1128/mbio.03721-21)
Supplement: FIG S2 [file mbio.03721-21-s0002.pdf]

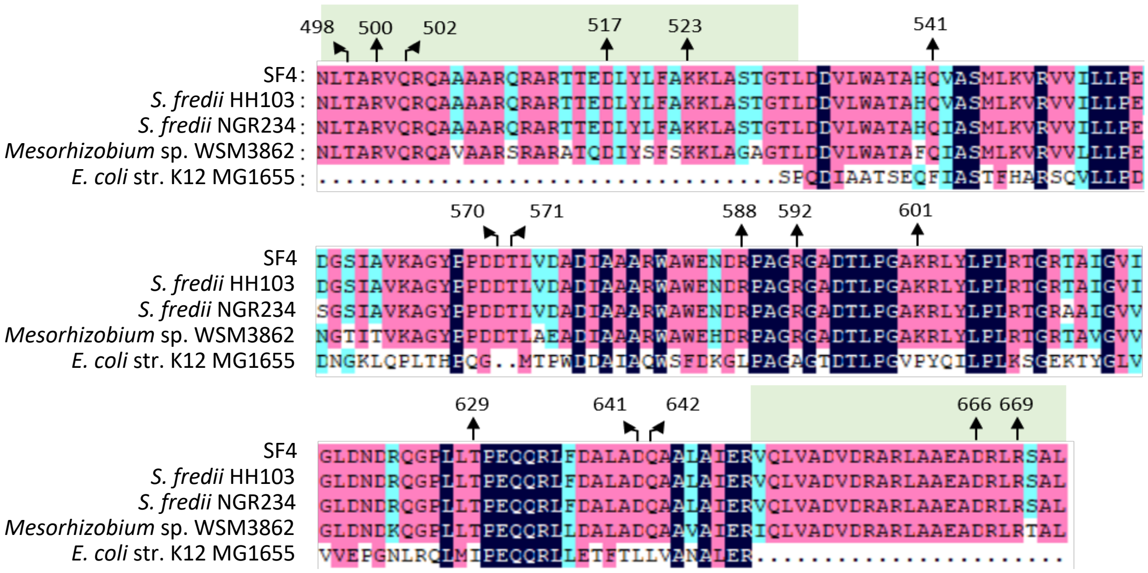

Fig. S2. Alignment of GAF domain of KdpD from representative rhizobia and *E. coli*. Polar residues subject to point mutation in Fig 6C are indicated. The green box shows regions absent in GAF of KdpD from *E. coli*. Identity levels are indicated in navy blue (100%), pink (75%) and azure (50%~75%).
