# Supplementary material for: The PTSNtr-KdpDE-KdpFABC Pathway Contributes to Low Potassium Stress Adaptation and Competitive Nodulation of Sinorhizobium fredii
Source: mBio. 2022 May 2;13(3):e03721-21. doi: 10.1128/mbio.03721-21 (PMC9239096; doi:10.1128/mbio.03721-21)
Supplement: FIG S3 [file mbio.03721-21-s0003.pdf]

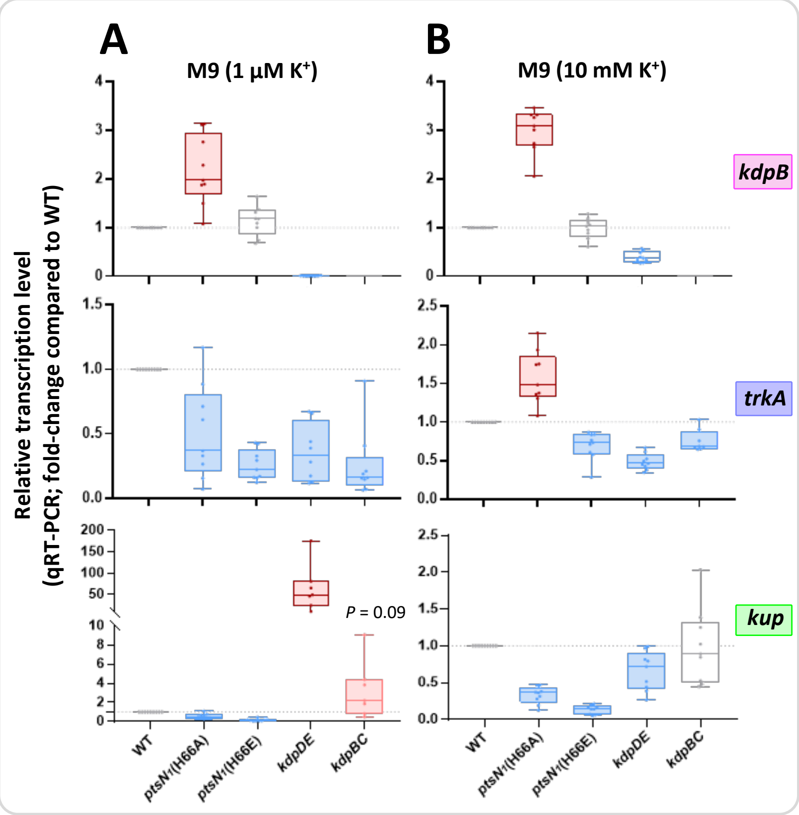

Fig. S3. Transcription profiles of potassium uptake systems in related mutants of the EIIA^{Ntr}-KdpDE-KdpFABC pathway. (**A**-**B**) qRT-PCR analysis of *kdpB*, *trkA* and *kup* genes in the *ptsN1(H66A)*, *ptsN1(H66E), kdpDE* and *kdpBC* mutants under 1 µM (**A**) and 10 mM (**B**) K^+ conditions in the M9 minimum medium. The reference gene is 16S rRNA gene. Significant difference is indicated based on one sample t test, theoretical mean = 1; red and blue represent significant up- and down-regulation, respectively, at $P < 0.05$ or at the marginal P values as indicated (three biological replicates with three technical replicates).
